# Combined effect of heat and corona charge on molecular delivery to a T cell line *in vitro*

**DOI:** 10.1371/journal.pone.0293035

**Published:** 2023-10-18

**Authors:** Molly A. Skinner, Alex Otten, Andrew Hoff, Mark Jaroszeski

**Affiliations:** 1 Department of Chemical, Biological, and Materials Engineering, University of South Florida, Tampa, FL, United States of America; 2 Department of Electrical Engineering, University of South Florida, Tampa, FL, United States of America; 3 Department of Medical Engineering University of South Florida, Tampa, FL, United States of America; Xiangtan University, CHINA

## Abstract

With the rapid increase of gene and immunotherapies for treating cancer, there is a need to efficiently transfect cells. Previous studies suggest that electrotransfer can provide a non-viral method for gene delivery. Electrotransfer traditionally relies upon the application of direct current pulses to the cells of interest. Corona charge was investigated in this study as an alternative to traditional methods as a means of creating the electric field necessary to deliver materials via electrotransfer. The goal was to determine if there was an increase in molecular delivery across the membrane of a human T cell line used as a model system. In a novel dish created for the study, the effects of elevated temperatures (37, 40, 43, and 45°C) during the treatment process were also examined in combination with corona charge application. Results showed that treating cells with corona charge at room temperature (~23°C) caused a statistically significant increase in molecular delivery while maintaining viability. Heat alone did not cause a statistically significant effect on molecular delivery. Combined corona charge treatment and heating resulted in a statistically significant increase on molecular delivery compared to controls that were only heated. Combined corona charge treatment and heating to all temperatures when compared to controls treated at room temperature, showed a statistically significant increase in molecular delivery.

## Introduction

There are two main classes of gene delivery methods: viral and non-viral. Viral methods use modified viruses to deliver a genetic payload. Non-viral methods typically use a chemical or physical mechanism to deliver genes rather than viruses [1]. Viral methods may yield effective transfection but they may also pose a safety hazard due to potential immunogenic response, mutation and toxicity [2]. Non-viral chemical methods primarily employ liposome based carriers because they are inexpensive and easily customizable [3], but they typically produce lower expression levels than viral methods [4]. Non-viral physical methods use physical force to puncture, weaken, and/or compromise the cell membrane to introduce new genetic material [5]. These methods are becoming more popular in gene delivery because they are also inexpensive and generally do not involve toxic or immunogenic materials. Some physical methods are non-invasive or minimally invasive; these include methods such as sonoporation and magnetoporation [5]. Electroporation, the creation of pores in the cell membrane due to an electric field, is an attractive physical delivery method as it does not have the same complications as viral or chemical methods [6, 7]. It has been shown to be effective for most cell types with a higher transfer efficiency than other physical methods [7]. *In vivo* and *in vitro* electroporation require metallic electrodes to be in contact with the cells/tissue being treated. Electroporation can be done easily on some tissues, but can become invasive or even impossible for deeper tissues due to the necessity for contact. Some gene therapy applications require an *in vitro* (or *ex vivo*) transfection, of which traditional electroporation methods my not optimal because of the highly localized currents causing unwanted effects such as cell damage [6, 7].

As mentioned previously, electroporation involves the application of direct current (DC) pulses to the cells using metallic electrodes. An alternative is to apply a flux of corona charge, the gathering of charged particles in a neutral fluid, instead of electrode-tissue-electrode DC pulses as it does not require contact between two metallic electrodes and the cells or tissues being treated [8]. Corona charge normally occurs when ions are created by applying an electrical potential to an electrode that has a sharp surface or point such as a needle with a nearby electrode, such as a plate, that is at a lower potential [8, 9]. This study investigated the application of the negative electrical potential to a plate electrode rather than the needle. It is hypothesized that corona charge can provide an alternative way to apply the electric current to cause cell membrane permeabilization *in vitro* to transfer materials across the cell membrane [10]. Cell suspensions were located on the plate electrode. This was done to attract the positive ions created at the needle electrode to the cells contained, in suspension, on the plate. This ensured that the ions would go through the cell suspension rather than remaining on the surface of the suspension. Corona charge utilized for this study was generated using a negatively charged plate and a needle that was located above, and not touching, the fluid containing cells. This corona charge is classified as non-thermal (cold) and was produced at atmospheric pressure in ambient air.

Plasma is similar to corona charge except that it is produced in a gas other than air; helium is a commonly used pure gas for creating plasma. Plasmas (including corona charge) are hypothesized to cause membrane permeabilization *in vitro*, enough to transfer genetic material, while still maintaining cell viability [10]. A number of studies followed that used helium plasmas [6, 11–18] and corona charge [7, 8, 19–21] to transfect cells. A majority of these studies used murine melanoma (B16.F10) cells or a human keratinocyte line (HaCaT). A 2008 study used both positive and negative corona charge to induce molecular delivery of multiple fluorescent molecules to a B16F10 cell line. This not only demonstrated that molecular delivery was significantly increased with the application of corona charge, but that negative and positive corona charged produced similar levels of molecular delivery and post-treatment viability [7]. Another study was conducted in 2015 using corona charge to transfect B16.F10 and HaCaT cells with luciferase *in vitro*. This showed that after 72 hours, there was a 5-fold increase in expression levels with no effect on cell viability [8].

The application of corona charge at ambient as well as increased temperatures was investigated because a 2014 study showed that heat had a positive effect on gene uptake in combination with traditional electroporation [22]. Consequently, the goal of this study was to determine if corona charge could increase molecular delivery to a human T cell line *ex vivo* alone and with heat. The motivation was to eventually improve upon primary T cell transfection for cell-based therapies.

## Materials and methods

### Cells, media, and tracer molecules

This study used Jurkat, Clone E6-1 cells (ATCC TIB-152, American Type Culture Collection, Manassas, VA), an immortalized human T lymphocyte cell line. This cell line is commonly used as a model human cell line in cancer therapy development [23]. They were cultured in RPMI 1640 1X with L-glutamine (11875093, Gibco, Grand Island, New York). Media was supplemented with 10% (v/v) Fetal Bovine Serum (Corning 35011CV, Corning Cellgro, New York, New York), 1% (v/v) 200 mM L-glutamine (25030081, Gibco), and 1% (v/v) penicillin-streptomycin (15140122, Gibco). The cells were seeded in 75 cm^2^ flasks (Corning 430641, Corning Cellgro) and grown in a standard 37°C incubator with a humified environment containing 5% CO_2_. Culture flasks were seeded with **~**2.8 million cells and supplemented with media for a total volume of 13 ml within the flask to produce a final concentration of 1.5 million cells / ml after 48 hours [24].

Cell populations were utilized for experimentation if they were at least 95% viable. Viability was determined using 0.4% trypan blue dye solution (15250061, Gibco, Grand Island, New York). They were prepared by first washing three times by centrifugation (200 RCF for 5 minutes) in Dulbecco’s phosphate-buffered saline with calcium and magnesium (PBS, 21-030-CV, Mediatech, Inc, Manassas, VA). Cells were suspended in PBS at a concentration of 2 million cells/ml for experimental use after washing [24].

SYTOX^TM^ Green Nucleic Acid Stain (S7020, Life Technologies, Eugene, Oregon), a fluorescent tracer molecule (963.58 Da), was used to quantify results. SYTOX^TM^ was used because when it binds to nucleic acids its fluorescence increases by more than 500-fold [11, 24] as compared to unbound SYTOX^TM^ in solution. Therefore, permeabilization of the membrane as a result of treatment with corona charge would allow SYTOX^TM^ to contact and bind to nucleic acids inside the cells. This produced a strong fluorescence signal that could be quantified using a microplate reader. In this study, a final concentration of 1 μM SYTOX^TM^ was used to detect molecular delivery by adding it immediately after treatment with corona charge and/or heat [12]. Viability using trypan blue was checked a few minutes post treatment to ensure that florescence was from delivery rather than cell death.

### Fluorescence measurement

**Fig 1:**
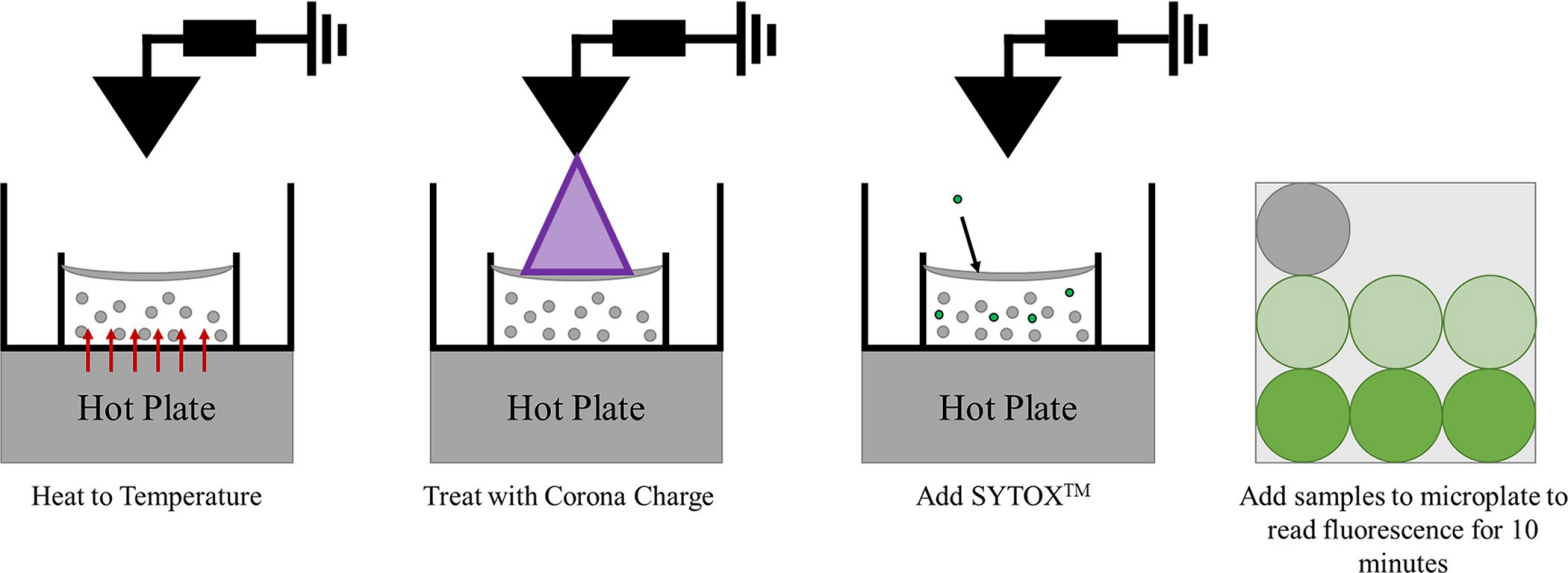
The steps taken to treat cells with corona charge to deliver SYTOX^TM^. 1) Heat cell suspension to temperature. 2) Treat with corona charge. 3) Add SYTOX^TM^ to cell suspension as soon as corona charge concluded. 4) Add samples to microplate a and fluorescence was read for 10 minutes.

Fluorescence data was acquired from paired samples, one control and one that was treated with a set of parameters. Three representative 150 μl aliquots of all samples were then pipetted into individual wells of a black polystyrene 96-well microplate (117017023, Corning Incorporated, Corning, NY). The plate was then placed into the BioTek FLx800 Microplate Fluorescence Reader (BT-FLX800T, BioTek, Winooski, VT). Fig 1 shows the procedural breakdown of the experimental process. Fluorescence was measured over a 10 minute period at 11 second intervals immediately after treatment, resulting in 56 measurement. More SYTOX^TM^ that was delivered to the cell interiors, resulted in a higher fluorescence reading. Viability was also measured to ensure that cell death was not the main cause for the change in fluorescence. The difference between mean fluorescence data at 10 minutes post-treatment and non-treated control samples was determined. The data at 10 minutes post treatment was chosen because the increasing fluorescent signal leveled off around that time for all samples. Differences between control and test samples were proportional to the quantity of delivered SYTOX^TM^ [24].

### Novel transfection dish design

**Fig 2:**
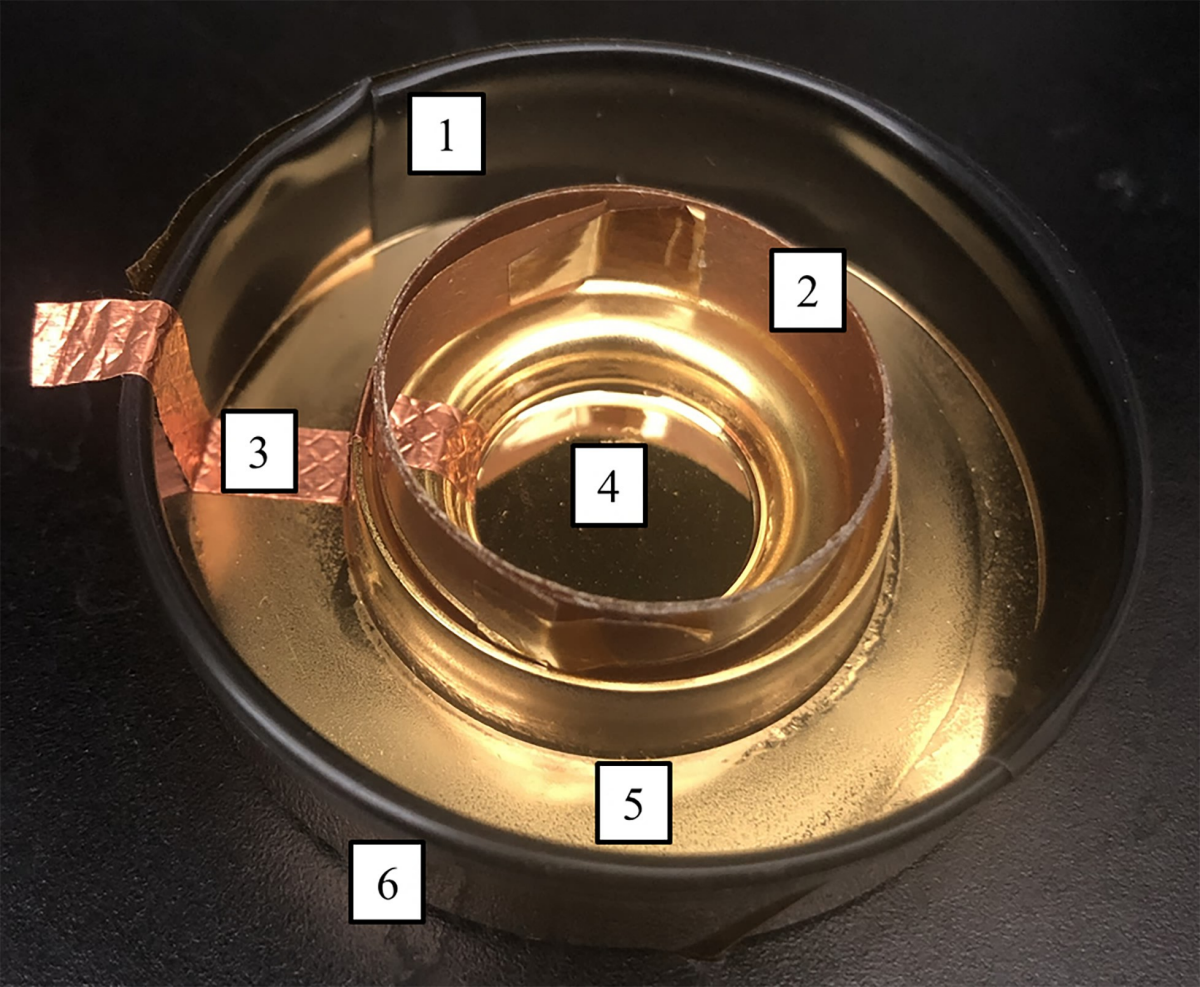
Modified cell culture dish used in experimentation. Made from a double well cell culture dish modified. 1) electrical tape, 2) Kapton, 3) copper tape, 4) center well where treatment occured (diameter of 18.1 mm and approximately, 1000 Å of gold over a layer of chrome), 5) matte enamel, and 6) Kapton tape [24].

Modified organ culture double-well dishes (Falcon 353037, Corning) were used for this study. These polystyrene dishes were modified by an initial sputter coating of a chromium followed by a 1000 Angstrom (Å) thick layer of gold. This made the surface of the dish electrically conductive. The culture dish was then coated in Matte Clear Enamel (7701830, Rust-Oleum, Vernon Hills, IL) to insulate the culture dish with the exception of the center well where cell suspension would ultimately be treated (indicated by 4 in Fig 2). Other insulating components included electrical tape, Kapton cylinder, and Kapton tape (indicated by 1, 2, and 6, respectively, in Fig 2). These measures helped direct charges to the cell suspension and prevent arching to other areas of the dish. Once the culture dish was well insulated, a piece of copper tape was placed so that it contacted the uninsulated cell treatment area and formed a pathway to the exterior of the culture dish. This piece of tape was connected to the high voltage power supply. Fig 2 shows an image of the culture dish with the specifications and modifications done to ensure insulation [24].

### Instrumentation

**Fig 3:**
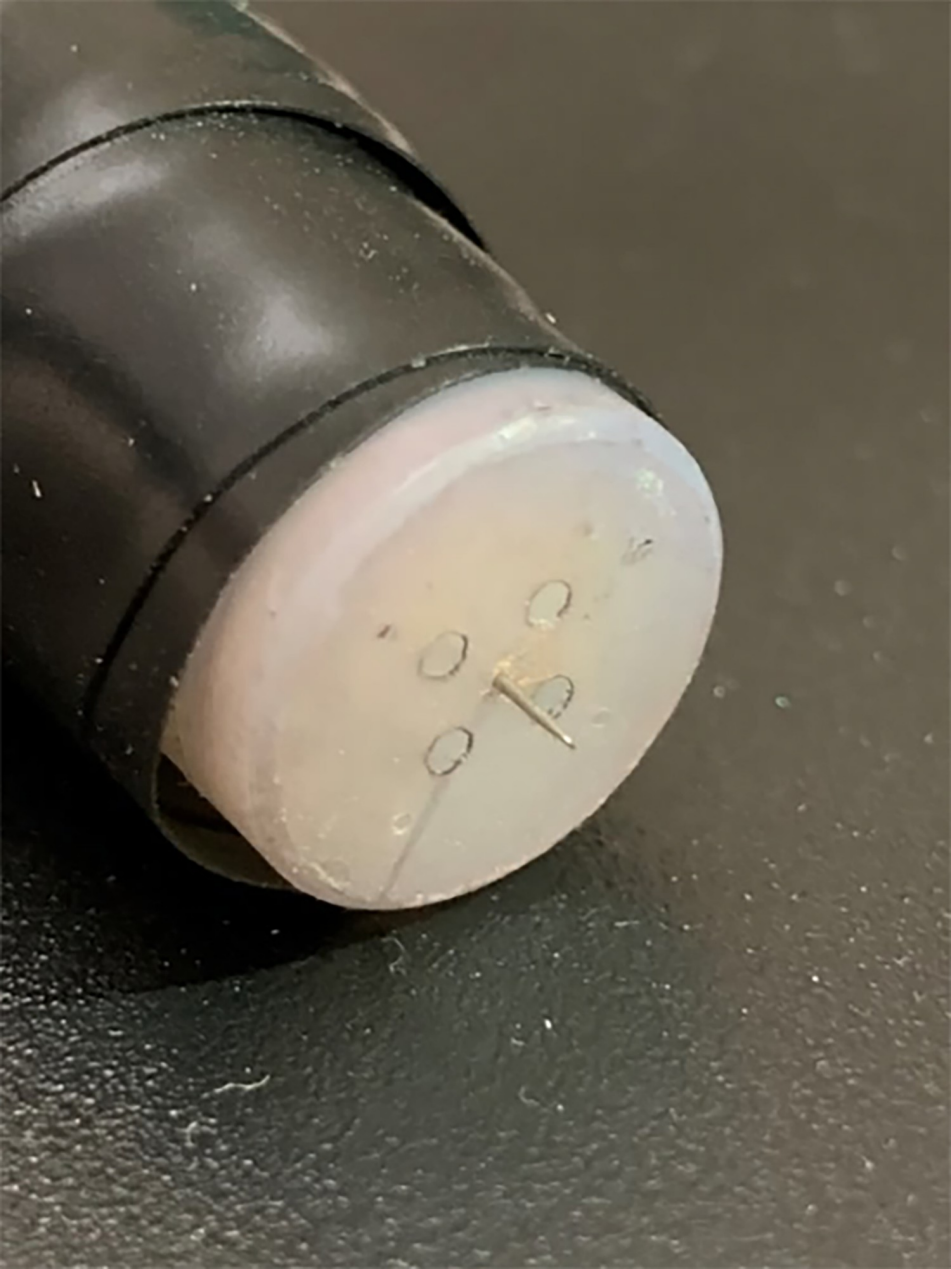
Single needle electrode / corona generator [24].

**Fig 4:**
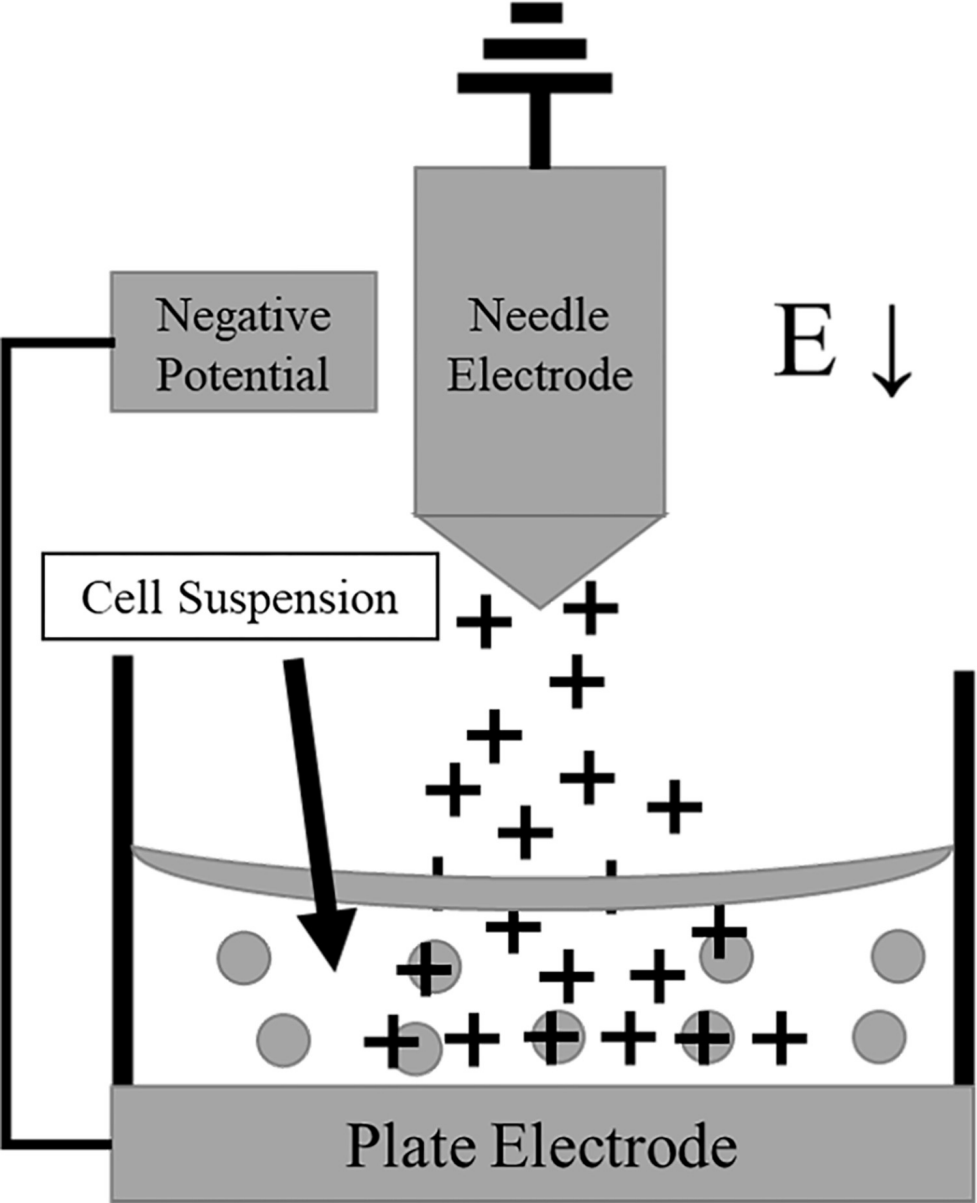
Physical orientation of the corona generator, cell suspension, and transfection dish. The electric field is pointing downward, sending the positive ions created at the needle electrode to the bottom of the transfection dish so they pass by the cell suspension.

Corona charge was generated using a single stainless-steel needle as an electrode (NA2840, Natural, China) exposed to the ambient atmosphere in a biological safety cabinet. The needle was 28-gauge with a body diameter of 350 μm and a tip diameter of 2 μm. The needle was held within a white Delrin® tube. The sharp tip protruded 3 mm from the end of the Delrin. Fig 3 shows the needle electrode [23]. Fig 4 diagrammatically shows a close up of the configuration of the needle over the dish.

**Fig 5:**
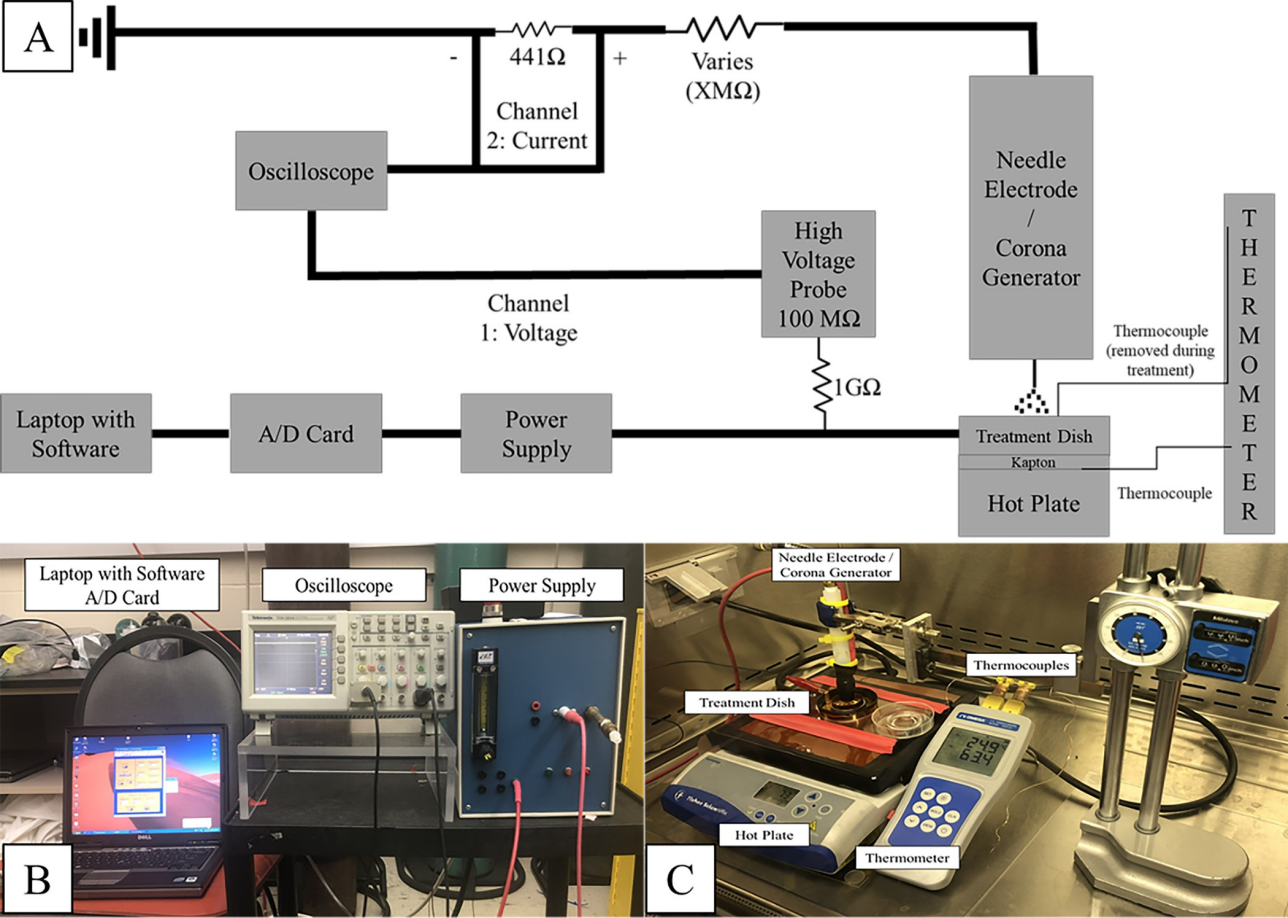
a) Schematic of apparatus used to control heat and corona charge parameters [24]. b) Labeled image of the laptop with software, A/D card, oscilloscope, and power supply. c) Labeled image of hot plate, treatment dish/plate electrode, thermometer, thermocouples, and needle electrode.

Fig 5 illustrates the schematic of the entire treatment system used in this study including the novel transfection dish and corona charge generator that are located on the right-hand side of the generator. The temperature of the Jurkat cell suspension within the transfection dish was controlled using a hotplate (309N0030, Fischer Scientific, Hampton, NH) that was located immediately beneath the transfection dish with a metal plate on top to ensure even heating. Temperature was an experimental variable measured by thermocouples (5SC-TT-K-30-36, Omega, Biel/Bienne, Switzerland). However, thermocouples have a metallic tip and could interfere with corona charge generation during treatment if they remained in the transfection dish/suspension. Consequently, they could not be used to monitor the temperature of the cell suspension during treatment. In order to ensure that the proper temperature was reached and maintained the hot plate was calibrated to achieve and maintain one of the four (37, 40, 43, and 45°C) desired temperatures for the suspension. To ensure that temperatures did not drift during treatment, a thermocouple was placed on the Kapton covered surface the transfection dish rested on. Since the temperature of the plate did not vary more than +/- 0.5°C, it was assumed the solution was within that tolerance as well. Calibration was done prior to each experiment to account for minor changes in atmosphere.

Corona charge was generated by applying DC voltage (- 10 kV) to the transfection dish using a power supply that was controlled using custom software, a laptop computer, and an analog to digital converting (A/D) card. Voltage and current were monitored using an oscilloscope, with voltage being measure directly and current being calculated by the voltage drop across a resistor (V = IR). The main difference from other systems that utilized corona charge and the system utilized in this study is that the connections were reversed. This means, for this study the high negative potential (voltage) was applied to the plate in the system and the needle was grounded. Typical systems, the connections are reversed, applying voltage to the needle, or an emitter, and grounding the plate. This was done in an attempt to create a flux of ions through the cell suspension by having the needle strip electrons from the air creating positive ions which are then attracted to the plate resting at a negative potential. The tip of the needle was positioned 3 mm above the liquid surface, creating a constant flux of positive ions near the needle. Ions were thus forced by the imposed field to transit from air to liquid and continue to the plate surface passing the cells in suspension.

### Treatment parameters

To determine parameters for delivery experiments, viability experiments were first performed on the cells. The viability of cells was an important aspect of this study to ensure metabolic activity so that cells could subsequently be expanded post-treatment in culture. For this reason, viability was investigated as a function of temperature and corona charge application independently. Parameters were selected if cell viability remained above 70% 48 hours after treatment. Temperatures of 45°C, 43°C, 40°C, and room temperature (~23°C) were investigated based on a previous study [22] which heated cells before treating with traditional electroporation. Human body temperature (37°C) was investigated later in this study [24]. Samples were heated to their respective temperature, then held at temperature for 5 minutes. The time it took to reach temperature varied between the different temperatures and there were slight differences due to environmental factors. To be certain that the cells were not damaged by hyperthermia (especially at 43°C and 45°C) at elevated temperatures, viability was assessed immediately after heating at hour 0 and again at hours 1, 2, 3, 4, 5, 24, and 48. It has been reported that human skin can be heated for 6 hours at 44°C before irreversible damage occurs. Between 44 and 51°C, the time to cause irreversible damage halves for every degree above 44°C [25, 26]. Given this data, it was likely that the Jurkat cells would survive heating to temperature for 5 minutes (post ramp-up time, which averaged ~2 minutes with minor changes from day to day), as the effects of hyperthermia would not take effect at an elevated temperature for that short of a time period.

All experiments involving corona charge were conducted using—10 kV and approximately 25 μA with the needle 3 mm above the liquid surface. This combination produced an acceptable viability level (>70% post-treatment). Each experiment used 700 μl of a cell suspension at a concentration of 2 million cells per ml. This translated to 1.4 million cells being used in the chamber for each sample and a liquid height of 2.75 mm.

### Statistics

All data comparisons were made using a Student’s one-tailed t-Test to determine if there was an increase in molecular delivery. The null hypothesis was that the values were not statistically greater. All comparisons were made using an n of 3 or greater and a level of significance (α) of 0.05 (a confidence level of 0.95). If the absolute value T_stat_ (calculated T-value) was greater than T_critical_, then the null hypothesis was rejected and there was a statistically significant increase between the two means [24].

## Results and discussion

### Effects of temperature and corona charge on cell viability

**Fig 6:**
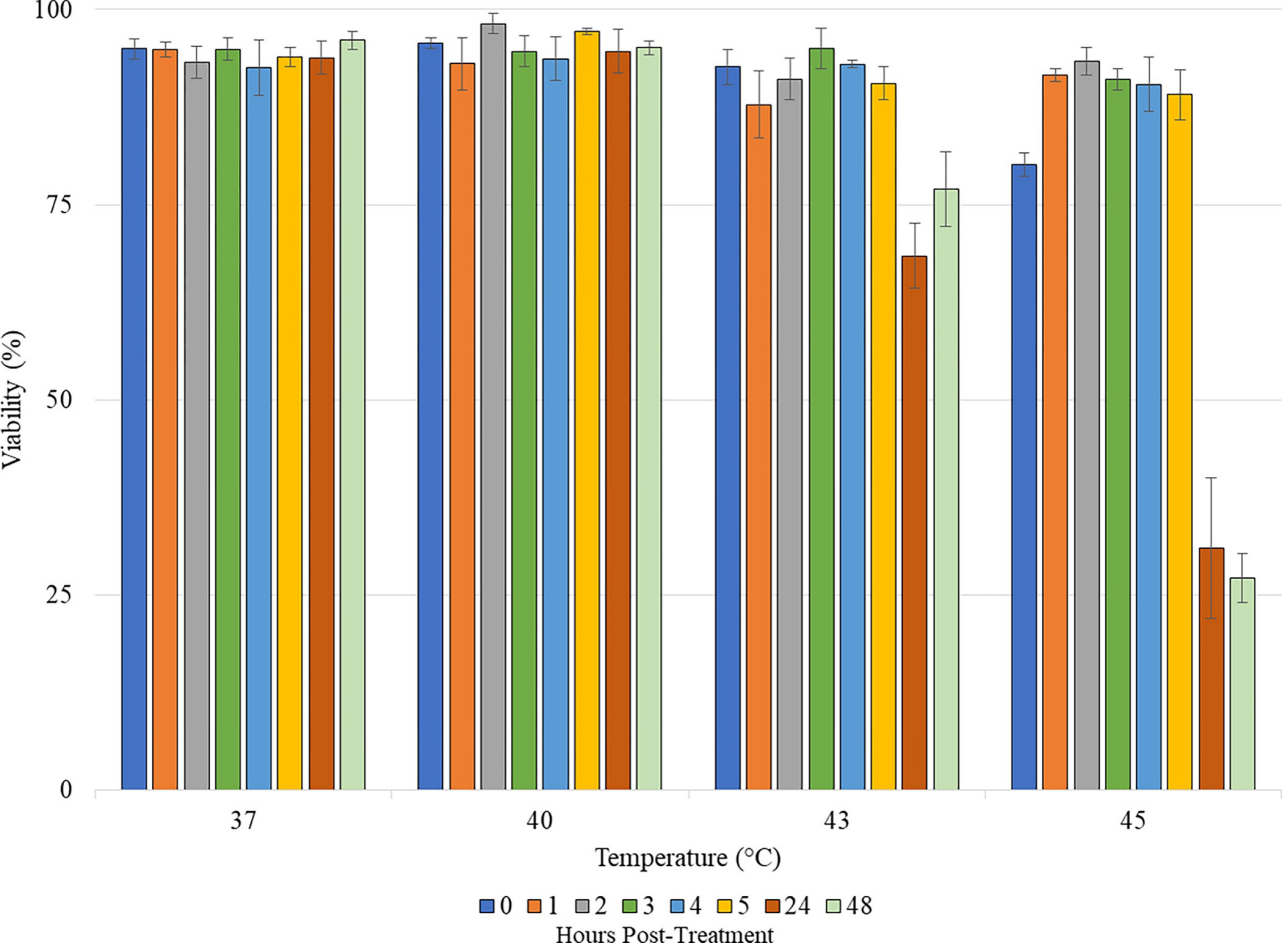
Viability of cells at different time points after heating to 45°C, 43°C, 40°C, and 37°C and holding temperature for 5 minutes. Each bar represents an average of 3 samples [24].

The effects of temperature on viability were determined using trypan blue at time 0 (immediately post-heating) and 1, 2, 3, 4, 5, 24, and 48 hours later as shown in Fig 6. Cells were incubated at 37°C with 5% CO_2_ during the 48 hour period. Viability for all temperatures remained above 90% for the first 5 hours. At 48 hours, viability for both 37°C and 40°C remained above 95%. For 43°C, the viability at 24 hours dropped, but by 48 hours, the viability increased above an acceptable level (>70%). For 45°C, the viability dropped drastically at 24 hours and further dropped at 48 hours. Cells could withstand being heated for up to 5 minutes at 37°C, 40°C, and 43°C, but not at 45°C, leading to 45°C to be eliminated from the parameter pool.

**Fig 7:**
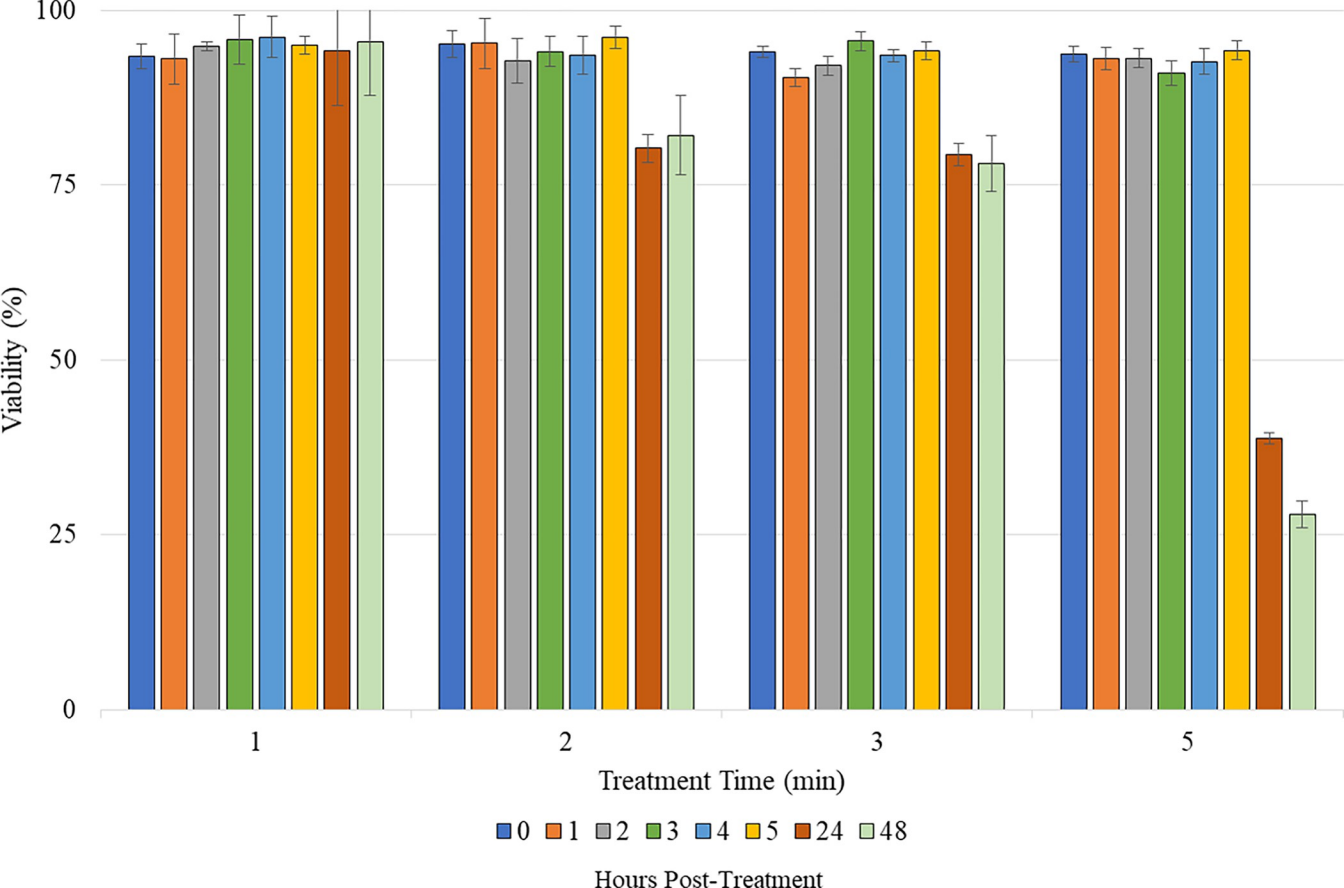
Viability of cells at different time points after treatment for 1, 2, 3, and 5 minutes with corona charge generated using -10 kV with 25 μA of current. Each bar represents an average of 3 samples [24].

Preliminary heat tests showed that cells could withstand being heated for up to 5 minutes with minimal impact on cell viability. The effects of corona charge (- 10 kV and 25 μA) on cell viability at treatment times of 5, 3, 2, and 1 minutes were determined next. These experiments were conducted at ambient temperature (~23°C). The results are shown in Fig 7. The viability at 24 and 48 hours after being treated with corona charge for 5 minutes treatment plummeted. There was a modest reduction in cell viability after 3 minutes of corona charge treatment. No effect on cell viability was observed when treating for 2 and 1 minutes. Based upon these results, the viability after treatment times of 1, 2, or 3 minutes remained high enough at hour 48 based on the standards set at the beginning of the study.

### Effects of corona charge treatment at ambient temperature on SYTOX^TM^ delivery

**Fig 8:**
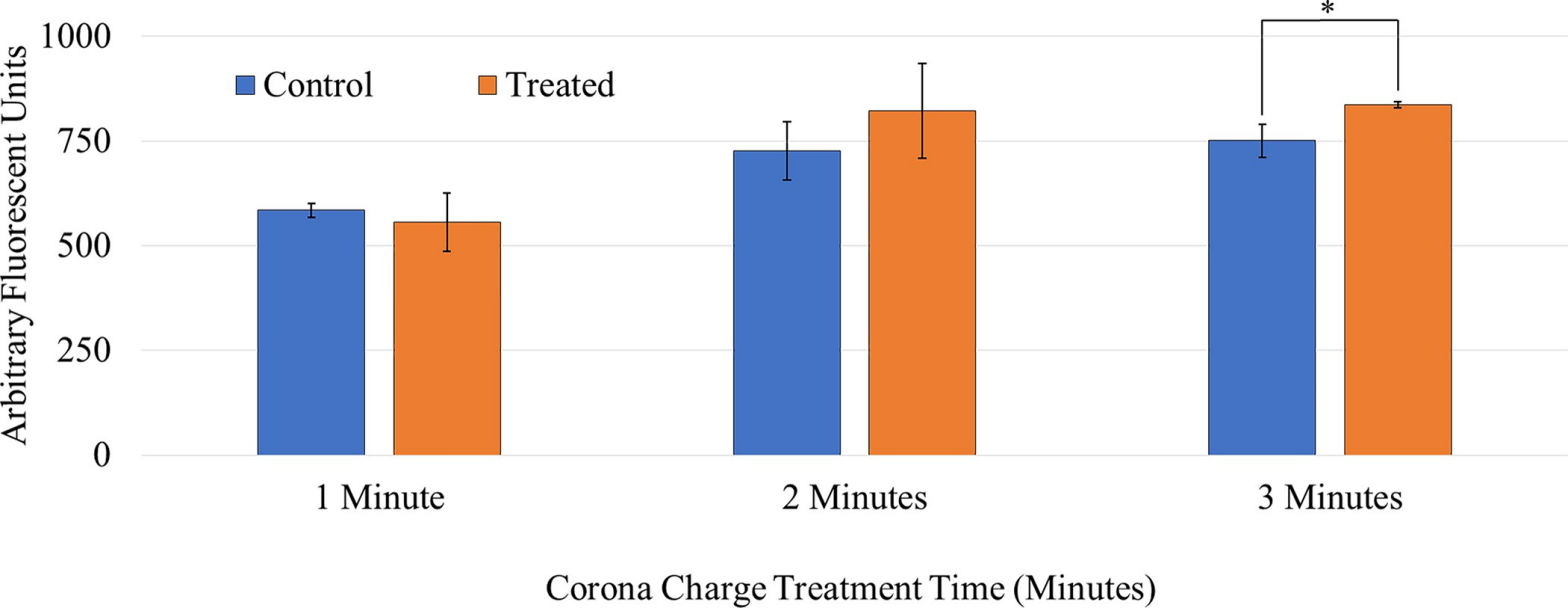
Effects of time on delivery of SYTOX^TM^ on corona treatment at ambient temperature. The * indicates statistical significance in the increase of the delivery of SYTOX^TM^. Each bar signifies an average of 3 samples with the error bars representing standard deviation [24].

**Fig 9:**
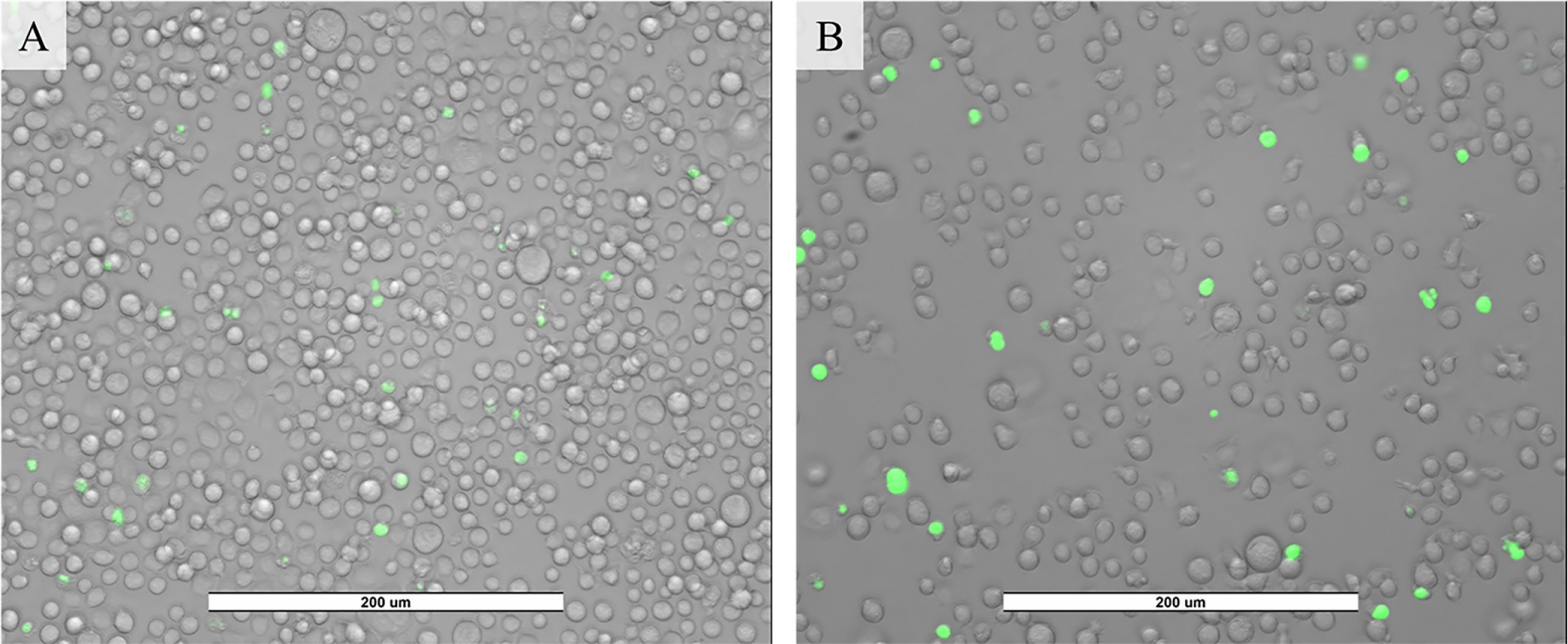
Images of Jurkat cells after SYTOX^TM^ delivery at -10 kV, ~25 uA, 3 minutes, and room temperature. A) Control: cells that have not been treated with corona charge with SYTOX^TM^ added. B) Sample: cells that have been treated with corona charge with SYTOX^TM^ added.

SYTOX^TM^ experiments were performed treating cells with corona charge for 1, 2, and 3 minutes at -10 kV and 25 μA to determine which treatment time would provide a statistically significant increase in uptake. Fluorescent data was taken 10 minutes post treatment to determine uptake. Fig 8 shows that treating for 1 and 2 minutes at ambient temperature did not cause a statistically significant difference in molecular delivery compared to samples that were not treated. Three minutes showed a statistically significant increase in molecular uptake. Therefore, subsequent experiments that involved corona charge treatment (with or without heat) were conducted using a 3-minute treatment time. Fig 9 shows microscopy images of SYTOX^TM^ delivery from applying corona charge at room temperature. It should be noted that SYTOX^TM^ signal can appear in cells for a few different reasons other than molecular uptake due to corona charge treatment. This caused a green signal in the control samples, as seen in Fig 9. The higher percentage of green positive cells and the higher intensity of the signal is evidence of molecular update due to corona charge treatment.

### Effects of heating alone on SYTOX^TM^ delivery

**Fig 10:**
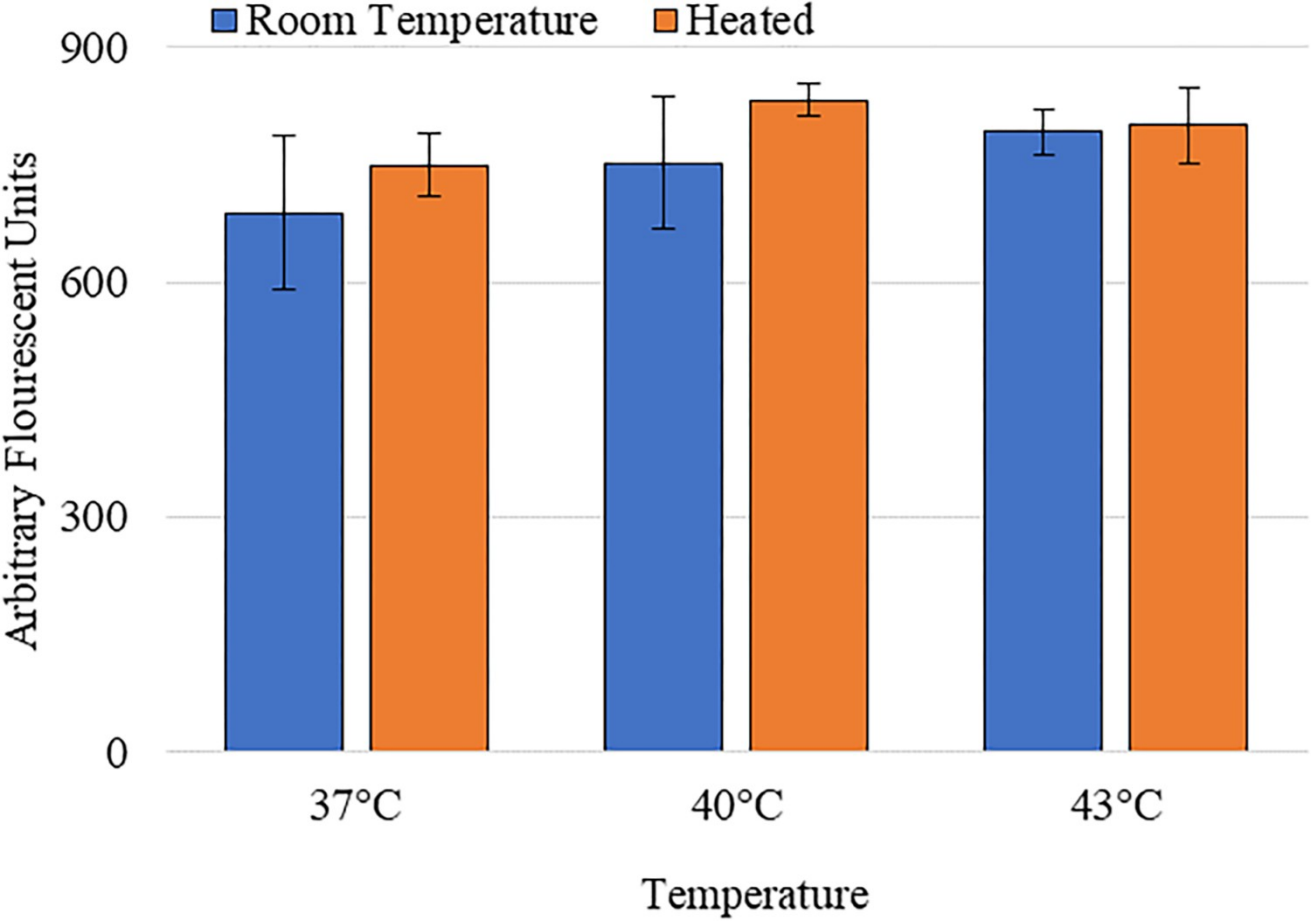
Effects of temperature increase alone on delivery of SYTOX^TM^ compared to room temperature. The * indicates statistical significance in the increase of the delivery of SYTOXTM. Each bar signifies an average of 3 samples with the error bars representing standard deviation [24].

The potential effects of heat alone on SYTOX^TM^ uptake were investigated. Control samples were maintained at room temperature (~23°C) and the experimental samples were heated to either 37°C, 40°C, or 43°C and held at temperature for 3 minutes to simulate being treated with corona charge. Fig 10 shows the difference of SYTOX^TM^ delivery between the control and the heated samples at each temperature. There was no statistically significant increase in the uptake of SYTOX^TM^ from heat alone.

### Effects of corona charge combined with heating on SYTOX^TM^ delivery

**Fig 11:**
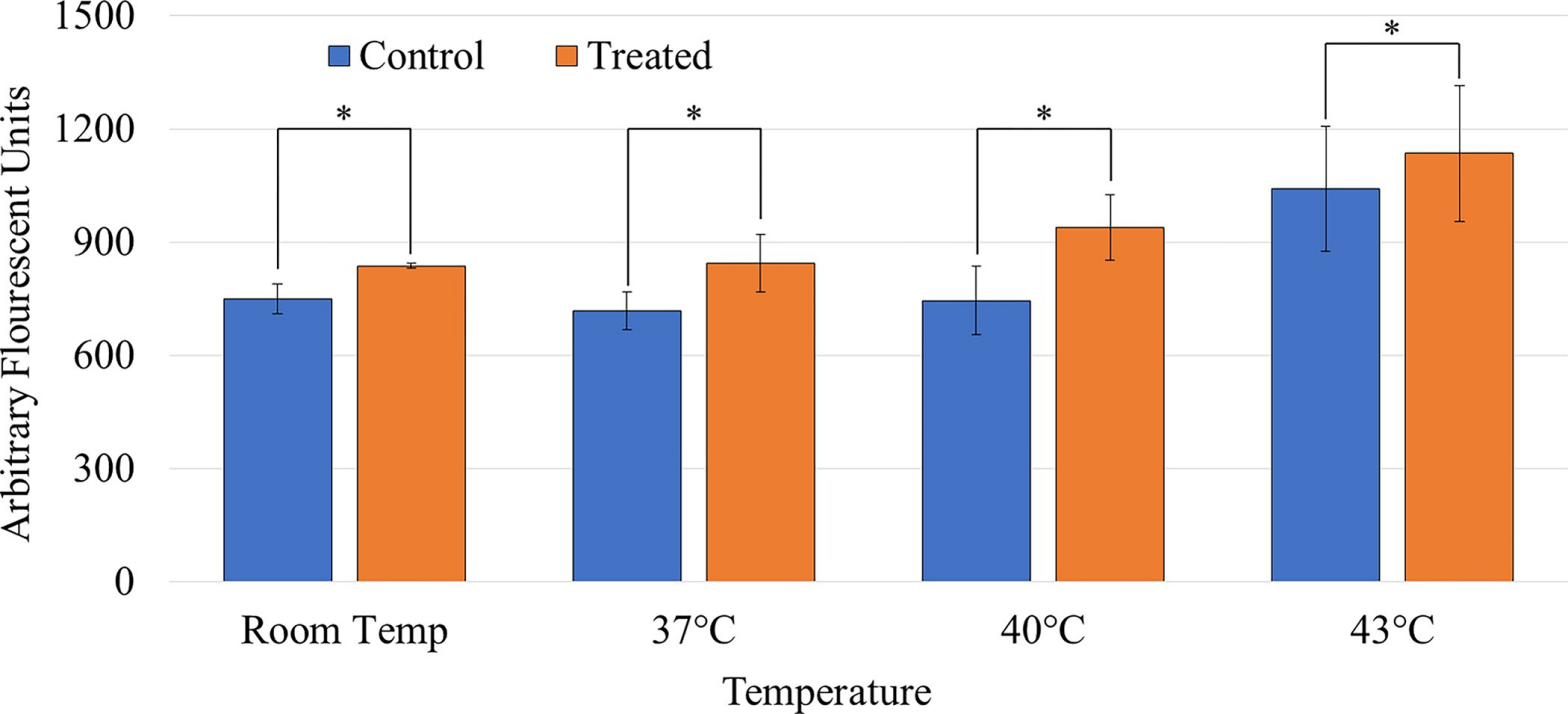
Effects of temperature increase and corona treatment on delivery of SYTOX^TM^ compared to control samples heated to temperature. The * indicates a statistically significant increase of the delivery of SYTOX^TM^. Each bar signifies an average of N samples with the error bars representing standard deviation. (N_RT_ = 3, N_37_ = 9, N_40_ = 14, and N_43_ = 12) [24].

At this point in the study, it was determined that a corona charge treatment time of 3 minutes would be used with an applied voltage of -10 kV and a current of 25 μA at 37°C, 40°C, or 43°C. Before treating cells with corona charge, they would first be heated to temperature and held at their respective temperatures for the 3-minute treatment time. Immediately after the corona charge treatment, SYTOX^TM^ was added to the cells and spectrofluorometric analysis was started. Controls samples were heated to temperature but were not treated with corona charge. Fig 11 shows the difference of SYTOX^TM^ delivery between the heated controls and treated and heated samples. All temperatures showed a statistically significant increase in the delivery of SYTOX^TM^. The average viabilities for samples at 37°C, 40°C, and 43°C were above 80% and average control viability of 93%.

**Fig 12:**
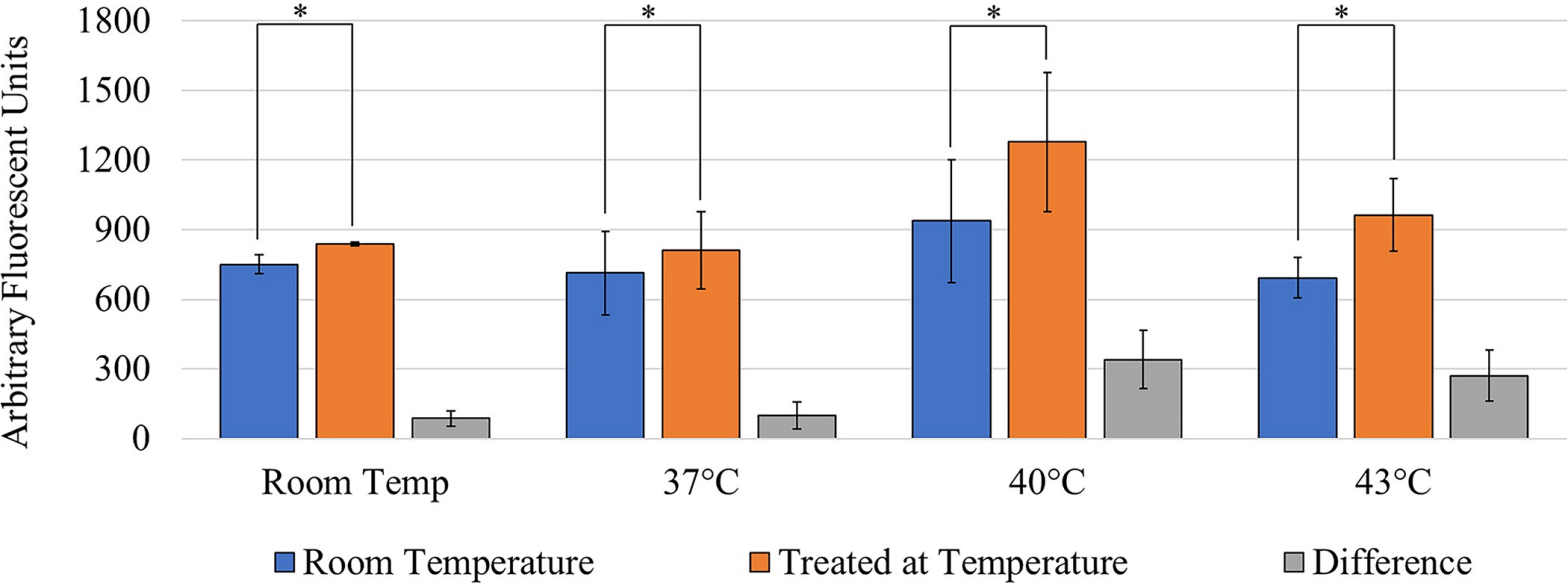
Effects of temperature increase and corona treatment on delivery of SYTOX^TM^ compared to room temperature, non-treated samples. The * indicates a statistically significant increase of the delivery of SYTOX^TM^. Each bar signifies an average of N samples with the error bars representing standard deviation. (N_RT_ = 3, N_37_ = 13, N_40_ = 9, and N_43_ = 9) [24].

A similar experiment was conducted where the control samples were kept at room temperature while the corona charged treated samples were heated to temperature. The data, shown in Fig 12, presents a statistically significant increase in molecular delivery between the control and samples. The viability for each temperature after treating was above 80% with an average control viability of 95%.

## Conclusions

The novel transfection dish created for this study was shown to deliver small molecules to Jurkat cells. While the transfection dish was designed to be reusable for experimentation, there are modifications that can be made to make it a single-use, disposable, and ready for mass production. This would eliminate the need for electroporation cuvettes where *in vitro* samples need to be pipetted in and out. The movement between a treatment and a growth surface can cause cell damage, decreases in numbers (due to cells being left behind), and viability decreases. The novel transfection dish design created for this study would allow cells to be treated and expanded in the same dish, decreasing the chances of cell damage and contamination from transferring treated cells. The system’s reversed electrical connections allowed the needle to collect electrons from the air to create ions that are attracted to the plate at a negative potential. This likely helped the corona ions to come into contact with the cells to induce electric field mediated uptake.

The optimal parameters were determined to be—10 kV, 25 μA, and 3 minutes at room temperature (~23°C), 37°C, 40°C, and 43°C. The corona charge and temperature parameters were selected because cell viability remained above 70% 48 hours after treatment. The delivery time was chosen because 3 minutes provided a statistically significant increase in SYTOX^TM^ delivery whereas 1-minute and 2-minute treatments did not. Prior to this study, it has been shown that corona charge can increase the molecular delivery of SYTOX^TM^ to murine melanoma cells [7]. It was also shown increase molecular delivery in Jurkat cells during this study at room temperature. Additionally, it was shown that heat alone could not deliver SYTOX^TM^ to Jurkat cells. The combination of heat and corona charge treatment demonstrated a statistically significant increase in SYTOX^TM^ delivery when compared to controls at room temperature and controls that were heated to the temperature at which the cells were being treated. The highest delivery was achieved at -10 kV, 25 μA, 3 minutes, and 40°C indicated by a highest fluorescent reading when compared to a room temperature control and a control held at 40°C.

In summary, this study demonstrated that molecular delivery may be achieved *in vitro* for a human T cell line with a corona charge system with reversed connections while still maintaining high viability post treatment [24]. This study delivered small molecules, however moving forward, many therapies rely upon the transfection of nucleic acids to T cells. Future studies will focus on determining the parameters necessary to deliver nucleic acids utilizing the system and the novel transfection dish, with minor adjustments for delivering a much larger molecule.

## Supporting information

S1 Raw data (XLSX)
